# Regulatory Patterns of a Large Family of Defensin-Like Genes Expressed in Nodules of *Medicago truncatula*

**DOI:** 10.1371/journal.pone.0060355

**Published:** 2013-04-01

**Authors:** Sumitha Nallu, Kevin A. T. Silverstein, Deborah A. Samac, Bruna Bucciarelli, Carroll P. Vance, Kathryn A. VandenBosch

**Affiliations:** 1 Department of Plant Biology, University of Minnesota, Saint Paul, Minnesota, United States of America; 2 US Department of Agriculture-Agricultural Research Service-Plant Science Research Unit, Saint Paul, Minnesota, United States of America; Instituto de Biología Molecular y Celular de Plantas, Spain

## Abstract

Root nodules are the symbiotic organ of legumes that house nitrogen-fixing bacteria. Many genes are specifically induced in nodules during the interactions between the host plant and symbiotic rhizobia. Information regarding the regulation of expression for most of these genes is lacking. One of the largest gene families expressed in the nodules of the model legume *Medicago truncatula* is the nodule cysteine-rich (*NCR*) group of defensin-like (*DEFL*) genes. We used a custom Affymetrix microarray to catalog the expression changes of 566 *NCR*s at different stages of nodule development. Additionally, bacterial mutants were used to understand the importance of the rhizobial partners in induction of *NCR*s. Expression of early *NCR*s was detected during the initial infection of rhizobia in nodules and expression continued as nodules became mature. Late *NCR*s were induced concomitantly with bacteroid development in the nodules. The induction of early and late *NCR*s was correlated with the number and morphology of rhizobia in the nodule. Conserved 41 to 50 bp motifs identified in the upstream 1,000 bp promoter regions of *NCR*s were required for promoter activity. These *cis*-element motifs were found to be unique to the *NCR* family among all annotated genes in the *M. truncatula* genome, although they contain sub-regions with clear similarity to known regulatory motifs involved in nodule-specific expression and temporal gene regulation.

## Introduction

In legumes, biological nitrogen fixation results from the mutualistic interaction of root cells with rhizobia in specialized organs called nodules [1]. This interaction leads to modification of gene expression in both host and bacteria [2]. Various techniques including mutant analysis, reverse genetics, suppressive subtractive hybridization, expressed sequence tag (EST) profiling, and macroarray and microarray gene expression analysis [3], [4] have been used to identify plant genes involved in nodule development and function in the model legume *Medicago truncatula,* hereafter referred to as Medicago.

In some legumes, a strikingly large number of genes encoding nodule cysteine-rich peptides (*NCR*s) are highly expressed in nodules. Expression of members of this family in nodules was first reported in *Pisum sativum* [5], followed by *Vicia faba* [6], Medicago [7], and *Galega orientalis* [8]. Three independent studies found that these *NCR*s appear to be legume-specific and are part of a large (>300 members) gene family [9], [10], [11]. Interestingly, no *NCR*s were identified in ESTs derived from nodules of *Glycine max* and *Lotus japonicus,* which led to the hypothesis that *NCR*s are specific to the inverted-repeat loss clade (IRLC) of legumes [10], [11].

Graham et al. [11] found *NCR*s to be similar to defensins in gene structure and genome organization. Defensins are a highly variable gene family found in vertebrates, invertebrates, plants, and fungi that have antimicrobial, anti-viral, and/or insecticidal activity [12], [13]. Plant defensins are highly conserved in sequence and are highly expressed in seeds with some family members expressed constitutively or induced by pathogen invasion [14], [15], [16]. Defensin-like (DEFL) proteins are a diverse superfamily that includes many additional classes of peptides in addition to the NCRs. All DEFL classes have a characteristic conserved pattern of cysteine residues and collectively include hundreds of gene family members in each of the sequenced plant genomes [17], [18]. Sizeable *DEFL* clades are characteristic of different plant lineages and are constitutively expressed in a tissue-specific manner [17], [18]. For example, in the Brassicaceae a large expansion occurred among reproductive tissue-specific *DEFL*s in the S-locus cysteine-rich (*SCR*) family [19] and pollen-tube chemoattractant *LURE*s [20]. In contrast, evidence for reproduction-regulating *DEFL*s in Medicago is scant and instead a large expansion occurred among the NCR class of DEFL peptides. Data from EST expression patterns [10], transcript profiling using the Affymetrix Medicago Genome Array [21], and a custom *DEFL* microarray [22] has added to the inventory of *NCR*s expressed in Medicago nodules, although information on the regulation and temporal expression patterns of most *NCR*s is lacking.

Recent work has begun to shed some light on the function of NCRs. A few NCR proteins were shown to be required to induce terminal differentiation of rhizobia [23]. In a complex dialog between host and symbiont, the rhizobial membrane protein BacA was shown to reduce the antimicrobial activity of specific NCR proteins, enabling development of nitrogen-fixing bacteroids to proceed [24]. BacA deficient mutant bacteria were killed rapidly upon challenge with these NCRs. However, these initial functional insights likely represent only a portion of the functional activity of this very large and diverse family.

We report a detailed study to identify mechanisms regulating expression of *NCR*s. We used a custom Affymetrix microarray with probes for 684 Medicago *DEFL*s to explore the expression patterns of *NCR*s in nodules inoculated with *Sinorhizobium meliloti* 1021 (Sm1021) at several developmental stages and nodules inoculated with mutants derived from Sm1021. Because wild type nodules do not develop synchronously, the mutants are helpful in dissecting the expression patterns within the nodules at different stages due to the arrested nodule growth at specific points in development. The mutants also provide information on the role of specific rhizobial components in inducing expression of *NCR*s. In tandem with the analysis of the expression patterns, we carried out an examination of the upstream 2,000 bp region of Medicago *NCR*s. Because *NCR*s are a large family of genes with different expression patterns, we hypothesized that specific DNA motifs present in the putative promoter sequences are associated with specific expression patterns and provide insights into the transcription factors that regulate their expression.

We found that 566 of the 684 Medicago *DEFL*s on the custom microarray were expressed in nodules at various stages of development. The 566 *NCR*s can be grouped into early *NCR*s and late *NCR*s based on their expression patterns and transcript abundance is dependent on the volume of rhizobia present in the nodule. The upstream 1,000 bp of the putative promoter regions of the *NCR*s have conserved DNA motifs that overlap with known *cis*-regulatory elements as well as novel motifs involved in nodule expression.

## Results

### *NCR* Expression Patterns are Dependent on Nodule Maturation and Rhizobial Development

**Figure 1:**
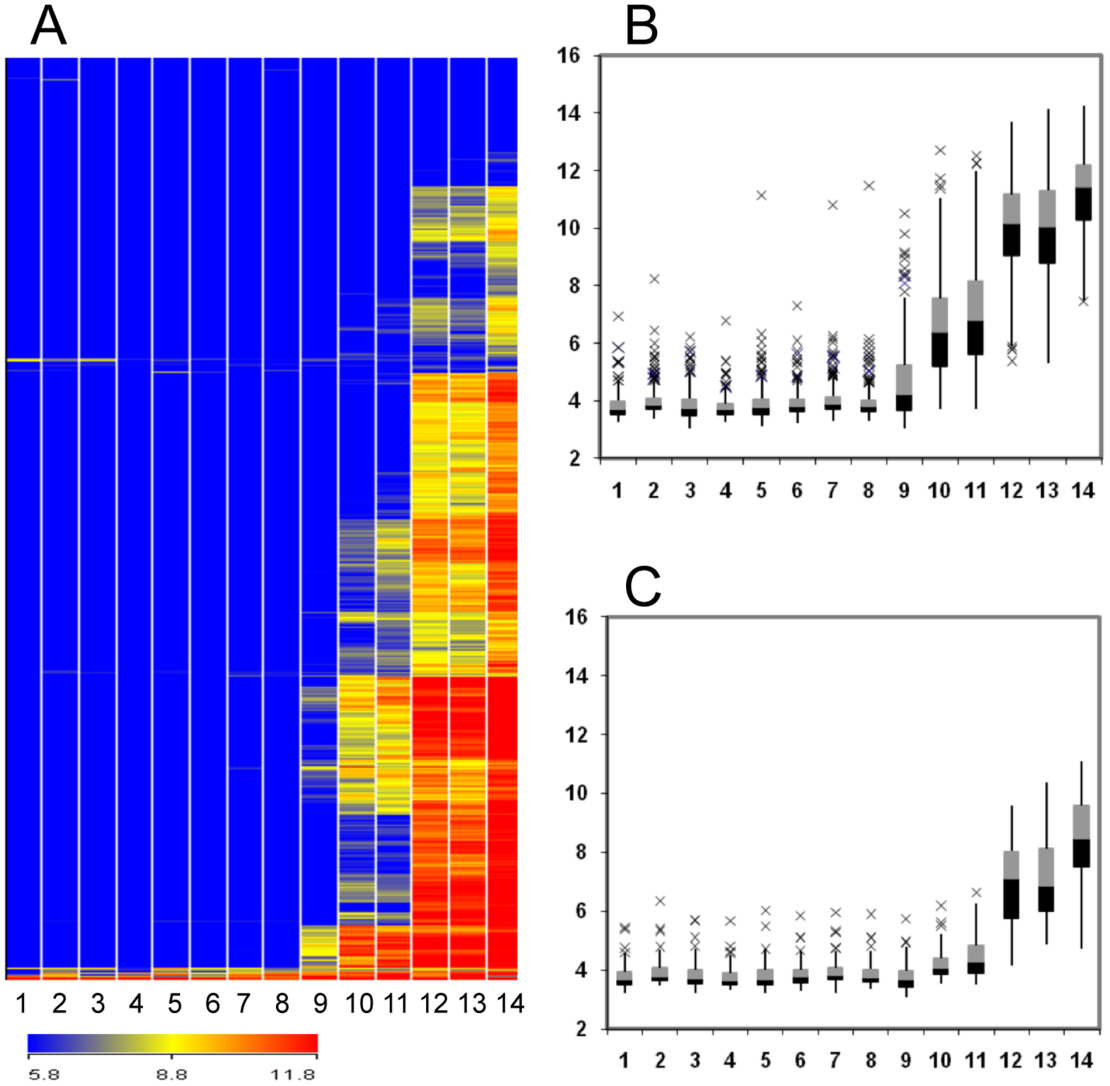
Expression profiles of *NCR*s. Expression values are log_2_-transformed intensity values. A, Hierarchical clustering (Euclidian average) of 571 NCR*s* and 14 treatments. Columns 1, 2, 3, 4, and 5 are data for mock-inoculated roots at 0, 4, 7, 14, and 40 dpi, respectively; columns 6, 7, 10, and 12 are roots inoculated with *S. meliloti* mutants *nodC*, *exoY*, *bacA* and *nifH* at 14 dpi, respectively; and columns 8, 9, 11, 13, and 14 are roots inoculated with Sm1021 at 3, 4, 7, 14, and 40 dpi, respectively. Color scales representing signal intensities are shown at the bottom. B, Expression profiles of 346 early *NCR*s. C, Expression profiles of 79 late *NCR*s. The box and whisker plots represent five different groups of intensity values, the minimum of which is the lowest whisker, the 25% quartile is represented by the bottom box, the 50% quartile is indicated by the median line, the 75% quartile is represented by the top box, the maximum value is the highest whisker, and the outliers are represented by x’s.

A custom microarray with probe sets for 684 Medicago *DEFL*s was used to identify *NCR*s, the subset of genes expressed in nodulated root fragments. This array was developed to identify gene expression patterns for the large family of Medicago *DEFL*s, many of which were identified computationally and for which expression data was lacking. In roots inoculated with Sm1021, infection threads penetrated the root cortex by 3 days post-inoculation (dpi) and proliferated within the nodule primordium by 4 dpi. Acetylene reduction assays indicated that the onset of nitrogen fixation occurred at 7 dpi (Figure S1). Nodules were fully mature at 14 dpi and by 40 dpi a senescence zone had formed. The *NCR*s that were differentially expressed during nodule maturation at 3, 4, 7, 14, and 40 dpi with Sm1021 are presented in Table S1. We used mock-inoculated roots at 0 dpi as a common control because very few (a total of 24 *NCR*s) were differentially expressed in roots over the time course covered by the study (Table S2, S3). During nodule development, the number of *NCR*s that were expressed increased from 15 *NCR*s in young nodules at 3 dpi to 527 in nodules at 40 dpi (Table 1, Figure 1A).

**Table 1:** Differentially expressed *NCR*s in nodules with different inoculations.

Nodule Stage^a^	Inoculum^b^	Number of Differentially Expressed Genes^c^	
		Positive	Negative
3 dpi	Sm1021	8	7
4 dpi	Sm1021	160	6
7 dpi	Sm1021	385	6
14 dpi	Sm1021	507	4
14 dpi	*nodC*	3	6
14 dpi	*exoY*	10	5
14 dpi	*bacA*	371	3
14 dpi	*nifH*	506	510
40 dpi	Sm1021	521	6

^a^Time point of nodule harvest.

b *S. meliloti* strain used for inoculation.

^c^Number of positively and negatively differentially expressed *NCR*s in comparison to mock-inoculated roots at 0 dpi. The *NCR*s with log_2_ fold-change >1 and with false discovery rate adjusted P<0.05 are classified as differentially expressed.

**Figure 2:**
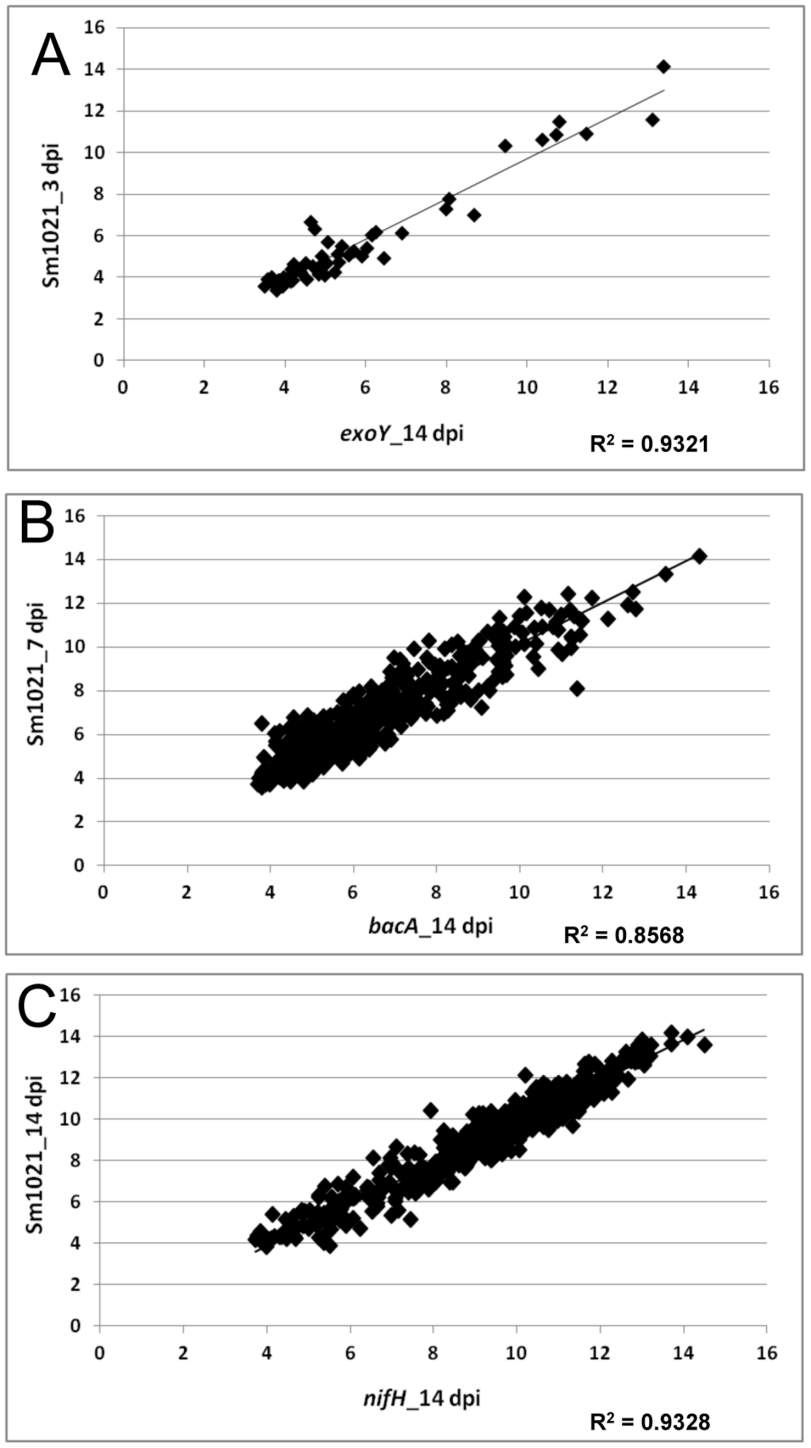
Correlation of *NCR* expression between nodules induced by Sm1021 and corresponding developmental time point mutants. All *NCR*s with “present” calls between the two treatments plotted were used to generate the scatter plots. Linear regression plots with a significant correlation (R^2^) are shown. A, Comparison between nodules induced by *exoY* at 14 dpi and Sm1021 at 3 dpi. B, Comparison between nodules induced by *bacA* at 14 dpi and Sm1021 at 7 dpi. C, Comparison between nodules induced by *nifH* at 14 dpi and Sm1021 at 14 dpi.

**Figure 3:**
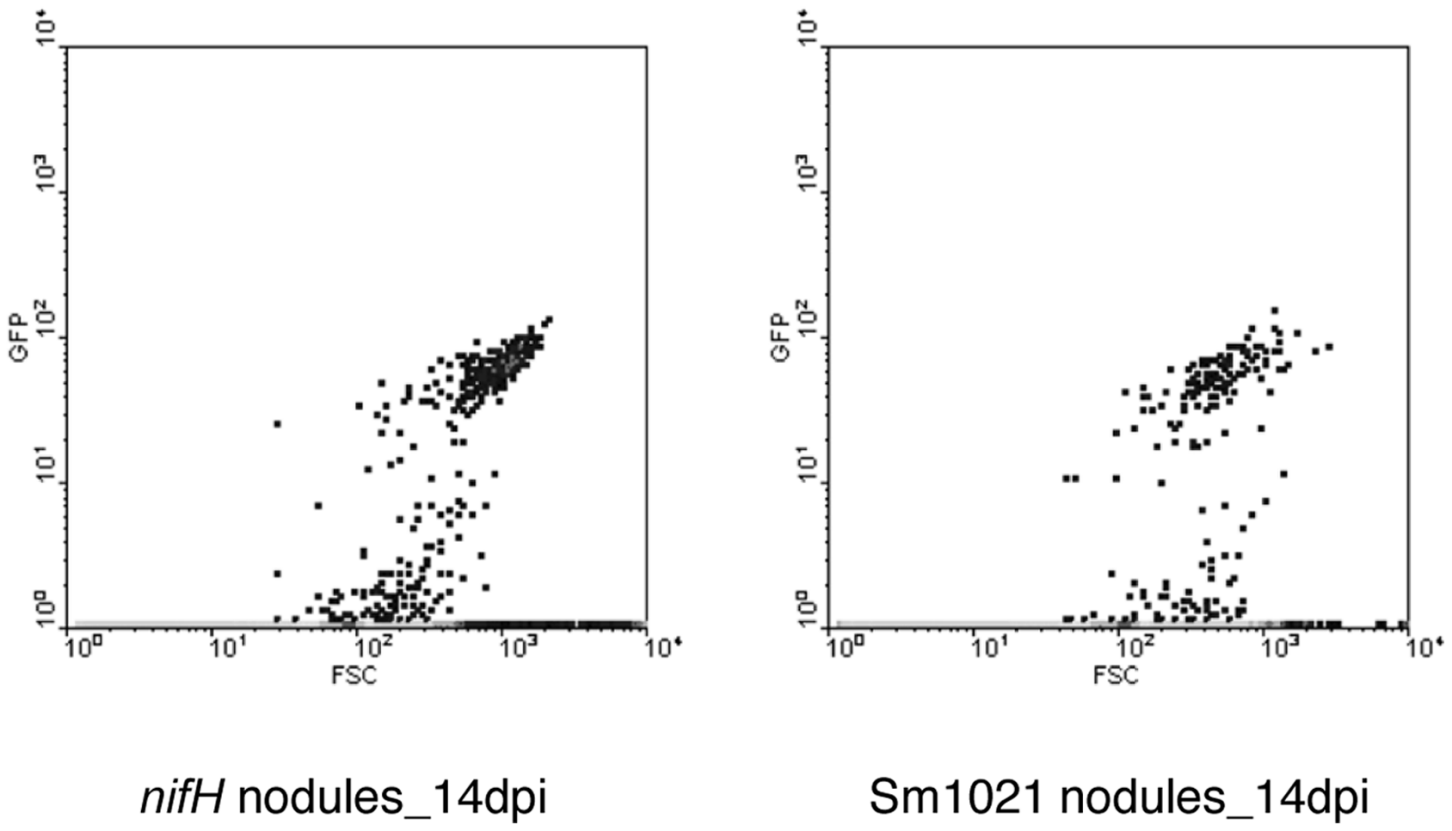
Quantification of bacteria in nodule extracts by flow cytometry. The density plots are based on fluorescence (GFP) detected against the rhizobial cell volume (Forward Scatter or FSC) in nodules formed 14 dpi with *nifH* or Sm1021. Twenty biological replicates (nodules from 20 different plants) were used for each treatment. The t-test p-value was >0.95.

We compared *NCR* expression in samples induced by rhizobial mutants with nodulated roots formed after inoculation with Sm1021. The *nodC* mutant cannot induce nodule formation because Nod factor synthesis is blocked [25], while the *exoY* mutant induces formation of nodules but nodules lack bacteria [26], and the *bacA* mutant induces nodules in which rhizobia senesce before differentiating into bacteroids [27]. Nodules formed after inoculation with the *nifH* mutant are deficient in nitrogen fixation [28]. Nodules formed after inoculation with bacterial mutants were harvested at 14 dpi for expression studies. Expression patterns in nodules blocked at different stages of development resembled expression patterns at different time points in the development of Sm1021 nodules. *NCR* expression in nodules induced by mutants *exoY, bacA,* and *nifH* at 14 dpi most closely resembled the expression in Sm1021 nodules at 3, 7, and 14 dpi, respectively (Figure 2, Table S4). Expression of *NCRs* in nodules formed by *nifH* and Sm1021 at 14 dpi was highly correlated (R^2^ = 0.93) suggesting similar *NCR* expression patterns in these two types of nodules. It has been previously reported that nodules formed by the *nifH* mutant closely resemble wild type nodules in structure and contain differentiated bacteroids [28]. Results from flow cytometry assays demonstrated that the numbers of rhizobia in the two types of nodules at 14 dpi were not significantly different (Figure 3).

The *NCR*s were divided into early and late groups based on expression in the nodules formed by the *bacA* mutant compared to wild type nodules. A total of 346 early *NCR*s were expressed in *bacA* nodules (Table S5). All the *NCR* genes were expressed at lower levels in nodules formed by *bacA* at 14 dpi in comparison to the Sm1021 nodules at 14 dpi, with the exception of one gene that exhibited slightly elevated expression in *bacA* nodules than in Sm1021 nodules (Table S5). The late *NCR* group was composed of a set of 79 genes that were expressed in Sm1021 nodules but not in *bacA* nodules (Table S5). Expression of most of the early *NCR*s was first detected in Sm1021 nodules at 4 dpi and transcript abundance gradually increased with nodule age (Figure 1B). In contrast, expression of late *NCR*s was first detected in nodules at 14 dpi and, like the early *NCR*s, transcript abundance increased in older nodules (40 dpi) (Figure1C).

The expression of *NCR*s increased with rhizobial development (Figure 1A, Table 1, Table S4). Few genes were expressed in *nodC*-inoculated roots in which nodule development was blocked or in *exoY* nodules that lacked bacteria. A subset of genes that were expressed in wild type nodules were expressed in *bacA* nodules lacking bacteriod formation. Among the mutants, *nifH* induced the highest level of *NCR* expression in terms of both numbers of genes expressed and transcript abundance. Expression profiles of *NCR*s in *nifH* nodules were not significantly different compared to Sm1021 nodules indicating that bacterial number and development rather than nitrogen fixation regulates *NCR* expression.

To further investigate the role of nitrogen fixation in *NCR* expression, we used previously published data [29] to compare expression of *NCR*s in *dnf1* (defective in nitrogen fixation) nodules to nodules on wild type plants. In the Medicago *dnf1* mutant, wild type rhizobia enter the nodule via infection threads but do not differentiate into bacteroids [30]. This is similar to the phenotype observed in nodules formed by the *bacA* rhizobial mutant [27]. The plant growth conditions in both the studies were similar. Of the 103 *NCR*s in the dataset, 85 were down-regulated in *dnf1* nodules at 7 dpi and none were up-regulated (Tables S3, S6). A similar pattern of expression of the 103 NCRs was observed in our study in *bacA* nodules at 14 dpi compared to wild type nodules at 14 dpi (Table S3, S6).

### Conserved Motifs Occur Uniquely in the Upstream Regions of *NCR*s

To search for common *cis-*regulatory elements among *NCR*s, we mapped the position of 209 *NCR*s onto the sequenced BACs from the Medicago genome sequencing project (Mt2.0) and scanned the region 1,000 bp upstream from the translation start site (Table S7) using the Multiple Em for Motif Elicitation algorithm (MEME) [31]. This approach identified five conserved motifs ranging in length from 41 to 50 bp that each occurred in more than half of the input sequences (Table 2 and Figure S2). Prior to the release of Mt2.0, MEME analysis of only 50 *NCR*s yielded identical motifs to the much larger set of 209 analyzed, indicating that convergence was achieved. The five conserved motifs occurred in the upstream 1,000 bp region and were especially densely clustered approximately 400 bp upstream relative to the putative translation start site. Previously, Medicago *DEFL*s were grouped into subgroups based on sequence similarity [18]. Table S8 lists the *NCR*s, the subgroups to which they belong, and the organization of motifs in each gene. Motifs in *NCR*s that belong to nodule-specific subgroups [18] have more significant E-values compared to motifs in *NCR*s belonging to subgroups that are also expressed in other parts of the plant, suggesting that some of the motifs regulate nodule-specific expression. MEME was used to search for conserved motifs in upstream regions from early and late *NCR*s but no additional motifs were identified, suggesting that both groups have similar motif patterns.

**Table 2:** The five conserved motifs found in the upstream regions of NCRs.

Motif^a^	Length of motif (bp)	Number of Sites^b^	E-value	Motif Sequence
1	41	146	3.4E−772	AYTWARTRWGYTAAARRGAYAAAYAYRAYAYATTRATRTAH
2	41	153	4.5E−665	TTTYYYYTWWCTATYAYGAAAGGYTAWAAWAWWAWWDAAWW
3	50	160	1.9E−648	ARVAWDDDHWTTWTWMMYTATAAARTGATMAAAYMAWWTTHTWTWTDTWA
4	41	112	1.2E−492	TWARAGAYATTTAAYAATDATTYTAATTTTADRAADRDTTT
5	41	92	9.9E−288	TWVMAMYYYHWAARTGCTAAAAAYWATTWAATTRWWKTWAR

a The motifs were identified using MEME, had the most significant E-values of all motifs identified and are represented in more than half of the input NCR sequences.

b Number of promoters in which the motif was present in NCR genes.

To search for additional conserved motifs outside of the 5′ upstream region, the introns and the putative 3′ untranslated regions (1,000 bp downstream from the translational stop site) were scanned for motifs using MEME but the analysis did not reveal any significant new motifs. In addition, these regions were scanned for the five conserved *NCR* motifs using Motif Alignment and Search Tool (MAST) [32] but the motifs were not found. The conserved *NCR* motifs were also absent from the upstream 2,000 bp, introns, and 3′ untranslated regions of the 88 *DEFL*s not expressed in nodules. Furthermore, these motifs were absent in the 1,000 bp upstream of 33,131 annotated genes in the Medicago genome sequence (Mt2.0), excluding the *NCR*s. Thus, this unique combination of five motifs is confined to the upstream 1,000 bp region and clustered in the 400 bp upstream regions of *NCR*s in Medicago suggesting they are involved in nodule-specific expression.

### Known Plant Regulatory Elements Resemble Components of the Conserved Motifs Found in *NCR*s

We used Clover (Cis-eLement OVERrepresentation) [33], an algorithm that detects both under- and over-represented DNA motifs using statistical models, to identify the occurrence of known elements in the upstream regions of the *NCR*s. The upstream 1,000 bp regions from 209 *NCR*s, 88 *DEFL*s that are not expressed in nodules, and 3,000 non-*DEFL* genes from Mt2.0 were searched for the occurrence of the 104 plant regulatory elements in the TRANSFAC database (release 12.1). The latter two groups were used as two separate background models for comparison to the *NCR*s. Six known elements were found to be over-represented in the 1,000 bp upstream regions of the *NCR*s (Table 3). No statistically significant under-represented *NCR* promoter motifs were identified.

**Table:** **Table 3.** Over-represented TRANSFAC elements in *NCR* promoters.

Identifier^a^	Accession^b^	p-value^c^	Reference
P$ID1_01	M01021	0	[40]
P$ARF_Q2	M00438	0	TRANSFAC Reports 2∶0002 (2001)
P$PBF_Q2	M01130	0	TRANSFAC Reports 103∶0001 (2006)
P$PBF_01	M00355	0	[44]
P$DOF2_01	M00353	0.08	[44]
P$AGL1_01	M01059	0.005	[45]

a Element identifier.

b TRANSFAC Accession number of the element.

c p-values obtained from randomizing the elements using the non-*DEFL* Mt2.0 genes as background sequence sets. All p-values were 0.0 when using non-nodule *DEFL*s as a background set.

In order to compare the five motifs specific to nodule expression with the previously identified elements from TRANSFAC, we used STAMP, a web tool that compares sequence similarities between DNA motifs [34] (http://www.benoslab.pitt.edu/stamp). Table 4 lists the E-values for the correlations between the *NCR* motifs and known *cis*-elements. Motifs 1 and 2, which are 41 bp long, exhibited a high correlation with six small known motifs (6 to 12 bp long) that mapped within the longer *NCR* motifs. Using STAMP, we extended our comparison of the five conserved *NCR* motifs to known *cis*-elements from the literature and the plant *cis*-acting regulatory DNA elements (PLACE 30.0) database [35]. As a result, we saw that Motif 3 exhibited a strong correlation with ELEMENT1GMLBC3 (S000319; PLACE 30.0), which has been found in the promoter region of leghemoglobin in *G. max* [36]. Motifs 1 and 4 have the CTCTTT or the NICE element motif 2 [37], [38], which have been shown to confer nodule specificity to *Srglb3,* the leghemoglobin gene from *Sesbania rostrata*. We did not find any significant correlation between motif 5 and the elements evaluated in this study.

**Table:** **Table 4.** Mapping of *cis*-elements onto the *NCR* motifs.

Element^a^	Motif^b^	Orientation^c^	E-value^d^	Consensus Sequence^e^
P$ID1_01	Motif 1	sense	3.17E−09	TTTGTCSYTWT
P$ARF_Q2	Motif 1	antisense	6.42E−05	YTTGTCTC
P$PBF_Q2	Motif 2	sense	3.99E−05	WAAAGG
P$PBF_01	Motif 2	sense	7.16E−04	NWNWAAAGNGN
P$DOF2_01	Motif 2	antisense	3.13E−02	NNNWAAAGCNN
P$AGL1_01	Motif 2	antisense	4.05E−04	NTTNCCWWAWNNGGWAAN
ELEMENT1GMLBC3	Motif 3	antisense	0.00E+00	GATATATTAATATTTTATTTTATA
NICE2	Motif 1	antisense	5.09E−08	TTGTCTCTT
NICE2	Motif 4	antisense	8.09E−08	TTGTCTCTT
CTCTTT	Motif 1	sense	1.11E−03	CTCTTT
CTCTTT	Motif 4	antisense	1.94E−03	CTCTTT

a Elements were mapped onto the five *NCR* motifs.

b Motifs generated by MEME.

c Orientation of elements with the *NCR* motifs on the sense or antisense strands.

d E-values of correlation.

e Consensus sequence of the elements.

### Upstream 1,000 bp Region is Required to Drive *NCR* Expression in Nodules

**Figure 4:**
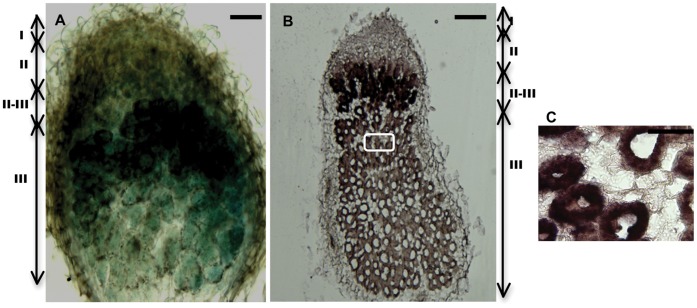
Localization of expression of an *NCR* in Medicago nodules. A, GUS staining of a nodule section with the MtTC100321_s_at promoter:GUS construct. B, Detection of the antisense probe corresponding to MtTC100321_s_at in a 10-µm thick nodule section. C, Magnification of boxed area in B. Bars in A and B are 200 µm. Bar in C is 100 µm. I, meristematic zone; II, infection zone; II-III, intermediate zone; III, nitrogen fixation zone of the nodule. All nodules were harvested at 14 dpi with Sm1021.

To investigate promoter function in more detail, we generated a promoter ß-glucuronidase (GUS) fusion construct using the 1,000 bp upstream region of the gene corresponding to probe set MtTC100321_s_at (Medtr3g062775, Medtr3g062810), an *NCR* with a very high transcript accumulation in nodules, and used it to generate transgenic Medicago roots. Histochemical staining for GUS activity revealed that this promoter is highly active in the interzone region (zone II-III) [39] with starch-rich cells where symbiosomes and bacteroids differentiate, and in the nitrogen-fixing zone (zone III) (Figure 4A). To determine whether the GUS expression pattern correctly reported the pattern of transcript accumulation, we hybridized an antisense mRNA probe of MtTC100321_s_at to nodule sections and found that the *in situ* hybridization pattern was similar to the pattern observed using the promoter::GUS fusion (Figure 4B, 4C). Earlier it was reported that transcripts of an early *NCR* (*NCR084*) mainly accumulated in the interzone II-III and expression of a late *NCR* (*NCR001*) occurred in zone III [10]. The gene corresponding to probe set MtTC100321_s_at is an early *NCR* with higher expression values compared to *NCR084* (MtTC94567_at; Medtr3g065710) in both early and late stages of nodule development. MtTC100321_s_at might have a different or extended functional role compared to *NCR084* and hence a different spatial localization pattern. In recent reports the late NCR peptides were observed only in the infected cells [23]. We also found that transcripts of MtTC100321_s_at accumulated only in infected cells in the nitrogen-fixing zone (Figure 4C).

**Figure 5:**
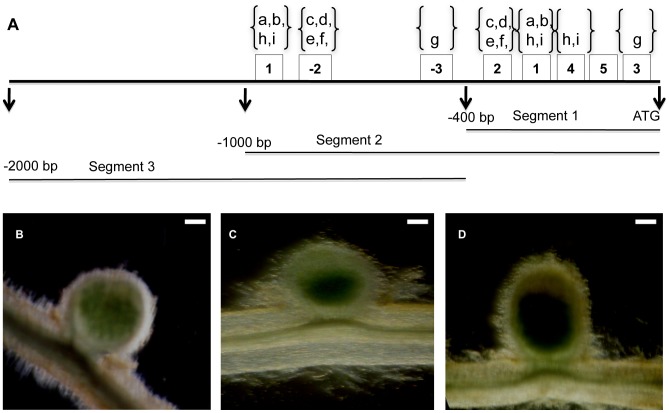
Promoter deletion assays. A, segments used for promoter deletion assays of gene corresponding to MtTC103606_at. 1, 2, 3, 4, and 5 are the conserved nodule motifs. The motifs found on the antisense strand are denoted as −2 and −3. The letters a through i designate the positions of elements P$ID1_01, P$ARF_Q2, P$PBF_Q2, P$PBF_01, P$DOF2_01, $AGL1_01, ELEMENT1GMLBC3, TTGTCTCTT, and CTCTTT, respectively. B, C, and D are transgenic nodules with constructs for GUS expression containing 1,000 bp upstream from the translation start site (segment 2) of genes corresponding to MtTC103606_at, MtTC95126_at, and MtTC100321_s_at, respectively. Nodules were stained at 14 dpi for GUS activity. Bars are 200 µm.

Because the upstream 400 bp region of the *NCR*s typically contained the five conserved *NCR* motifs, we performed promoter deletion assays to test whether this region was sufficient for promoter activity. Three *NCR*s were chosen for this assay. The genes corresponding to MtTC103606_at (Medtr5g076255) and MtTC95126_at (Medtr3g015870) were selected as representative of the most highly conserved promoter motif pattern, significant E-values with known *cis*-elements, and intermediate expression in mature nodules. MtTC100321_s_at was selected because the 400 bp region had a less typical pattern of motifs. We used three segments of the promoter region upstream of the translation start site to construct transformation vectors: segment 1 was 0 to approximately −400 bp from the start site, segment 2 was 0 to −1,000 bp from the start site, and segment 3 was −400 to −2,000 bp from the start site (Figure 5A). GUS expression was observed only in nodules with constructs containing segment 2 (Figures 5B–D, Figure S3), indicating that the 400 bp region upstream of the translation start site is necessary, but not sufficient, to drive gene expression in nodules. GUS histochemical staining intensity in the nodules tended to correlate with the expression levels from the MtDEFL chip data. MtTC100321_s_at, which has one of the highest expression levels of all the genes represented on the chip, exhibits the strongest GUS staining among the three genes (Figure S3).

### NCRs are Redundant in Function

Five different *NCR*s were selected based on their expression profiles and sequences for functional analysis based on the data from the custom *DEFL* array. MtTC100321_s_at is an early *NCR* with the highest level of expression in nodules at 14 dpi. It is also one of the few *NCR*s up-regulated in roots following infection with the pathogen *Phytophthora medicaginis*. Genes corresponding to MtTC100264_at, MtTC94214_x_at, MtTC108430_at, (Medtr3g069830) and MtAW775198_at (Medtr2g058625) were also selected for functional assays. MtTC100264_at and MtTC94214_x_at are expressed constitutively in vegetative tissues and nodules. MtTC100264_at belongs to the subgroup of classic Medicago-specific defensins and MtTC94214_x_at is a member of a defensin subgroup [18]. MtTC108430_at and MtAW775198_at are late *NCR*s and are only expressed in nodules. Each gene was individually knocked down using interfering RNA (RNAi) expression and over-expressed under the control of the *cassava vein mosaic virus* promoter. Plants were assayed for root and nodulation phenotypes with and without Sm1021 inoculation. Knock-down or over-expression of single genes did not result in an observable phenotype under our conditions. In particular, the ratios of bacteria to bacteroids in nodules on transgenic and control roots were not significantly different (Figure S4) and susceptibility of roots to *P. medicaginis* with RNAi and over-expression constructs was similar to controls. Quantitative RT-PCR confirmed the effectiveness of knock-down and over-expression (Figure S5).

## Discussion

### Expression of *NCR*s is Dependent on Nodule Development and Volume Occupied by Rhizobia in the Nodules

Based on expression patterns, *NCR*s can be divided into two major groups: early and late genes. Our data suggest that expression of the early group of *NCR*s was induced after the invasion of bacteria into nodules. First, we observed minimal *NCR* expression at 3 dpi in wild type nodules with significant expression at 4 dpi. Under our experimental conditions, infection threads proliferated extensively into nodule primordia at 4 dpi compared to 3 dpi. Secondly, minimal *NCR* expression was observed after inoculation with *S. meliloti* mutants *nodC* and *exoY.* The *nodC* mutant does not produce Nod factor, and therefore does not trigger deformation of root hairs or cortical cell divisions, thus no nodules form after *nodC* inoculation [25]. The *exoY* mutant elicits an early host response including the formation of young nodules; however, infection threads do not develop due to a defect in succinoglycan production [2], [26]. This suggests that the early *NCR*s are induced concomitantly with proliferation of infection threads. The early *NCR*s continued to be expressed in subsequent nodule developmental stages, with expression increasing as the nodule developed and rhizobia spread.

Transcripts corresponding to late *NCR*s were detected following bacteroid formation. Expression of late *NCR*s was low in wild type nodules at 7 dpi and significantly higher at 14 dpi. Expression of late *NCR*s following bacteroid formation is also supported by the lack of late NCR expression in the *dnf1* plant mutant and in *bacA*-induced nodules. The *dnf1* mutant and wild type plants inoculated with *bacA* form nodules in which bacterial differentiation is arrested soon after their release from infection threads resulting in nodules that are incapable of fixing nitrogen [30]. In both cases bacteroids do not form in nodules and contain low rhizobial populations.

*NCR* expression was also correlated with rhizobial numbers. Nodules of *M. truncatula* have an indeterminant growth pattern with a persistent meristem that remains approximately constant in size as the nodule matures. As new meristematic cells are produced, bacteria invade post-mitotic cells and subsequently both rhizobia and host cells begin to differentiate to support nitrogen fixation. The differentiated layers that harbor bacteroids increase in size during indeterminate growth [39]. We observed that *NCR* expression is induced when there is an extensive release of rhizobia from infection threads into infected cells at 4 dpi. The number of genes expressed and transcript accumulation increases as rhizobia differentiate and occupy a greater volume of the nodule. In the sparsely populated nodules formed after inoculation with *bacA* and or in the plant mutant *dnf1*, the number of *NCR*s expressed is low and expression is weak. This curtailment of expression may result when the plant detects disruption of nodule development and halts production of proteins required for late nodule differentiation, as has been seen for the late nodulins *MtLB1* and *MtCAM1*, which are not expressed in either *bacA* nor *dnf1* nodules [40]. The results of our experiments showed that *NCR* expression is not dependent upon nitrogen fixation. *NCR* expression patterns were similar in wild type nodules and nodules infected with *nifH,* which are similar in size and development to wild type nodules but are incapable of nitrogen fixation [28]. Also, similar numbers of rhizobia were found in nodules infected by *nifH* and in wild type nodules at 14 dpi, supporting our conclusion that *NCR* expression is dependent on the number of bacteria present and/or the volume occupied by them in the nodule. Further support comes from recent reports that surveyed the patterns of genes expressed in nodules at different developmental stages [4], [41]. In these studies, expression of the *NCR*s surveyed is higher in mature nodules filled with bacteria (>10 dpi) compared to incipient nodules (4 dpi).

### Regulation of *NCR* Expression in Nodules

In a previous study, we identified small, conserved, tandem duplicated sequences (mini-repeats) immediately upstream of the predicted translational start site of several *NCR*s that were clustered on the same BAC [11]. The five conserved motifs found in the current study in the upstream 400 bp of *NCR*s overlapped the regions of those mini-repeats. These five conserved motifs also contain previously known *cis-*regulatory elements that can be divided into two groups on the basis of their regulatory role.

The first group of elements includes the gene expression regulator ID1 binding site, Auxin Response Factor (ARF) binding site, Dof protein binding site, and MADS box genes binding site. ID1, a zinc finger transcription factor, binds to an 11-bp domain present in the promoter region of genes and regulates their expression [42]. An ID1-like transcription factor has been shown to be up-regulated in the Medicago*/S. meliloti* interaction [43] and may be involved in regulation of *NCR*s. Auxin is involved in regulation of gene transcription through the binding of ARFs to the *cis*-element (TGTCTC) present in the promoters of auxin response genes [44]. In the Medicago*/S. meliloti* symbiosis, auxin is involved in the initiation of nodule primordia and regulation of nodule number [45]. The presence of ARF elements in the upstream region of *NCR* genes suggests that auxin may contribute to regulation of *NCR* transcription in nodules. Dof proteins such as Dof2 and PBF are transcription factors unique to plants. They are known to bind to diverse plant promoters and have been speculated to participate in regulation of genes involved in photosynthesis and defense mechanisms, seed specific genes, and an oncogene [46]. MADS box genes are transcription factors found in both plants and animals. In plants they have been shown to regulate flower development [47]. In Medicago, transcripts of nodule-specific MADS box genes are localized in the infected cells and are probably involved in regulation of nodule-specific genes [48]. Additionally, based on data reported in the *Medicago truncatula* Gene Expression Atlas (MTGEA) (http://mtgea.noble.org/v2/), ID1, ARF, Dof, and MADS box genes are expressed in nodules of Medicago and ID1 in particular exhibits an expression profile that is highly correlated with *NCR*s.

The second group includes elements involved in nodule-specific gene expression, including ELEMENT1GMLBC3, CTCTTT, and NICE2, responsible for nodule-specific expression of leghemoglobin and a few nodulins [36], [37], [38]. Motif patterns of *NCR*s with early and late expression patterns were similar, suggesting that regulation of timing of *NCR* gene expression is complex. It would not be surprising that the large family of *NCR*s, which as a group has varied expression patterns and expression levels, should be regulated by a variety of transcription factors during the different stages of nodule development. The presence of common motifs among *NCR*s and leghemoglobin genes suggests that these regulatory motifs might have been recruited by *NCR*s from the more ancient nodule-specific leghemoglobins during the proliferation of *NCR*s that occurred after the divergence of the IRLC clade from other papillionoid legumes [49]. Further studies, including evaluation of the genome structure of *NCR*s, are required to test this hypothesis.

We identified five conserved motifs specific to nodule expression in *NCR*s, generally clustered in the region 400 bp upstream of the translation start site. Our promoter deletion assays showed that the 400 bp upstream segment tested was not sufficient to drive GUS expression, although it contained core promoter elements such as a TATA element, while GUS was expressed from the 1,000 bp upstream segment. Constructs with the 1,600 bp segment upstream of the 400 bp segment that lack the conserved motifs did not result in GUS expression. This suggests that the 400 bp upstream region with clustered motifs is not sufficient, but may be required to drive *NCR* expression in nodules. Although we did not find any signatures of the known core promoter elements in the −400 bp to −1000 bp segment, our results indicate that this segment contains additional motifs required for expression, possibly the additional copies of the conserved motifs observed in the −400 bp to −1000 bp segment. Further detailed analysis of the upstream 1,000 bp region using nested deletions or site-directed mutagenesis would be expected to reveal the significance of each motif in the regulation of *NCR*s.

### Possible Roles of *NCR*s

IRLC legumes, including *M. truncatula, P. sativum,* and *V. faba,* have indeterminate nodules with elongated, terminally differentiated bacteria with an amplified genome. Where sequence data are available, legumes in the IRLC are known to have *NCR*s [50]. Outside the IRLC, determinate nodule-forming legumes such as *L. japonicus* and *G. max* have nodules with rhizobia that do not undergo changes in cell and genome size and that can reproduce within the nodule. *L. japonicus* and *G. max* lack *NCR*s. It has been recently reported that a few NCRs have a lethal effect on free-living rhizobia in *in vitro* assays [23]. Similar to reports on defensin activity [51], these NCRs induce membrane modifications and inhibit bacterial cytokinesis. When one of the NCR was expressed in *L. japonicus,* it resulted in terminal bacteroid differentiation [23]. Host sanctions have been reported in *G. max* to prevail over “cheating” rhizobia, which take up carbon resources from the host without fixing nitrogen. Such host sanctions are yet to be reported in the IRLC legumes [52]. It has been suggested that the *NCR* family may have been recruited by the IRLC legumes to overcome the cheating mechanisms of rhizoba [23].

Here, we found that *NCR* expression levels correlate with the number of rhizobia present in the nodule. Very little is known about perception of rhizobial signal molecules after the initial perception of Nod factor. However, our results suggest that perception of rhizobial surface components or other signal molecules by the plant may trigger *NCR* expression. Recently, it has been shown that the rhizobial membrane protein BacA plays an important role in protecting rhizobia against the antimicrobial activity of some NCRs [24]. It has previously been reported that *BacA* expression in nodules is strongest in the II-III interzone where bacteria have been released from infection threads and complete their differentiation into bacteroids and it becomes weaker in the symbiotic zone with mature bacteroids [27]. Based on these observations, we speculate that as levels of BacA diminish in older infected cells, diverse NCRs accumulate to high levels in mature nodules and may ultimately be functional against rhizobia once the level of BacA drops below a critical threshold, triggering bacteroid senescence.

*NCR*s are classified into 35 subgroups based on sequence similarity [18]. Due to their large numbers and sequence diversity, it is possible that they are involved in multiple functions in nodules. Recent reports demonstrate that some plant defensins and DEFLs can function as signal molecules in plant development [53], regulation of reproduction [54], pollen tube development and guidance [20], [55], and pollen-stigma self-incompatibility interactions [19], [56]. Similarly, we hypothesize that the NCRs could themselves act as signals during nodule development. Additionally, because of their sequence similarity to defensins, they could be acting as anti-microbial peptides acting against the plethora of soil pathogens, as previously suggested [11].

We speculate that NCRs may have multiple roles to play in this complex network of communiqué between the microbe and the plant. DEFLs have been reported to play dual roles in defense and developmental signaling of plants [57], and so there is a possibility for an NCR to have more than one function. In whatever role(s) the NCRs are involved, our reverse genetic assays suggest functional redundancy among the many NCRs. A detailed study of molecular interactions between the host-microbe components, their regulatory factors and selection pressures is required to understand this large, fast-evolving, redundant family of genes.

## Materials and Methods

### Plant Material and Growth Conditions

Seeds of *M. truncatula* accession A17 were sterilized and germinated as described previously [58]. Seedlings were grown on buffered nodulation medium (BNM) [59], pH 6.5, solidified with 1.2% plant tissue culture grade agar (Sigma-Aldrich, St. Louis, MO) in 245 mm×245 mm plates (Corning, Lowell, MA). The radicles of sterile germinated seedlings were placed on moist, sterile germination paper on top of the agar medium and plates were wrapped with a sterile black cotton cloth (Cotton Club Black, #074300603820, Wal-Mart). Plates were placed vertically in a growth chamber with a 16 h photoperiod, 25°C daytime temperature, 21°C nighttime temperature, light intensity of 200 to 300 µmol m^−2^ s^−1^, and 50% relative humidity. At 5 d after planting, plants were inoculated with 100 µL/root of a washed suspension of *S. meliloti* 1021 (Sm1021, OD_600_ = 0.05) in sterile water. Control plants were mock inoculated with 100 µL/root with sterile water. For nodules and mock-inoculated roots harvested 40 dpi, the germinated seeds were planted in six-inch pots containing Turface (Profile Products LLC, Buffalo Grove, IL) and inoculated with Sm1021 as described above. Inoculated plants were fertilized once a week with aeroponic nutrient medium (LIPM formula) [60] without nitrogen while the mock-inoculated plants received nutrient medium with nitrogen. At 3, 4, 7, 14, and 40 dpi approximately 5 cm-long nodule-bearing root segments were harvested from inoculated plants and approximately 5 cm-long roots segments corresponding to the regions harvested from inoculated plants were collected from mock-inoculated plants. Root tips were removed at the time of harvest to eliminate transcripts from meristematic cells. Root segments were collected from three biological replicates, with samples collected and pooled from multiple plants in each replicate. Samples were immediately frozen in liquid nitrogen and were stored at −80°C for subsequent RNA extraction. Total RNA was extracted using TRIZOL reagent (Invitrogen, Carlsbad, CA) following the manufacturer’s instructions. During the RNA extraction, contaminating genomic DNA was removed by incubating samples with TURBO™ DNase following standard procedures suggested by the supplier (Applied Biosystems, Foster City, CA). The integrity and quality of total RNA was verified using the Agilent 2100 Bioanalyzer RNA 6000 Nano LabChip (Agilent Technologies, Santa Clara, CA). For all Medicago samples, 10 µg of total RNA was used to produce biotin-labeled cRNA using Affymetrix suggested procedures for 1-cycle eukaryotic reactions (Affymetrix). Ten micrograms of biotin-labeled cRNA, fragmented as suggested by Affymetrix, was hybridized to a custom Affymetrix microarray, the AtMtDEFL array [22]. The array includes 684 probe sets representing all previously identified Medicago *DEFL*s [18] as well as marker genes and invariant genes. The integrity and quality of labeled and fragmented biotin-labeled cRNA was verified using the Agilent 2100 Bioanalyzer RNA 6000 Nano LabChip. Arrays were hybridized, washed, stained, and scanned as previously described [61].

### Microarray Data Analysis

Data were normalized across the different nodule treatments using SBQ normalization (unpublished data) and validated across different treatments using quantitative RT-PCR (Figure S6). Differentially expressed genes were identified using the Empirical Bayes method within the LIMMA package distributed with R/Bioconductor. Both a 2-fold change cutoff and a Benjamini-Hochberg False Discovery Rate correction (P<0.05) were applied. The mean expression levels of the 566 NCRs across all the treatments with cross references to genes of Mt3.5v5 and genes identified by Mergaert et al. [10] is presented in Table S3. All microarray data in this study has been deposited in the Gene Expression Omnibus under accession number GSE34803: Expression data of Nodule Cysteine-Rich (NCR) Defensin-Like (DEFL) genes in different stages of nodule development in *Medicago truncatula* (http://www.ncbi.nlm.nih.gov/geo/query/acc.cgi?acc=GSE34803).

### Characterization of Nodule Phenotypes

For measuring nitrogenase activity, acetylene reduction assays [62] were performed using plants grown on BNM as described above at 6, 7, and 8 dpi with Sm1021. Five plants from each time point were blotted dry and placed one each in a 250 ml sealed jar. Twenty-five milliliters of air was withdrawn and replaced with an equal amount of acetylene. After 1 h, a 1 mL air sample was withdrawn and injected into a Photovac 10S Plus Gas C chromatograph (Photovac, Waltham, MA). Nitrogenase activity was expressed as nmoles ethylene h^−1^ plant^−1^.

The number of bacterial cells was measured in nodules from plants grown on BNM at 14 dpi with either Sm1021 or *nifH*. The bacterial suspension was prepared as previously described [63] and quantified on a Becton Dickson FACScalibur (BD Biosciences, San Jose, CA). Student's t-test was used to determine significant differences in bacterial populations between the two treatments.

### Identification and Analysis of Conserved Motifs

*NCR*s were mapped onto the Medicago genome assembly Mt2.0 using PASA [64]. For each gene four regions were extracted using custom Perl scripts: 1,000 bp upstream of the translation start site; 2,000 to 1,000 bp upstream of the transcription start site; introns; and 1,000 bp downstream of the translation stop codon. For motif discovery, several runs with different parameters of a locally installed MEME algorithm [31] were executed to find the best possible motifs using the module selecting 0 or 1 motif per site. MAST [32] was used to scan for the presence of motif models generated by MEME in the extracted regions of *NCR*s, *DEFL*s not expressed in nodules, and the 33,131 non-*DEFL* genes identified in Mt2.0. Sequences with E-values <10^−3^ were considered significant.

To scan for known elements in the 1,000 bp upstream regions of the *NCR*s, 104 plant motif matrices were extracted from the TRANSFAC® 12.1 database (http://www.gene-regulation.de/) and a locally installed Clover algorithm [33] was used to identify over-represented motifs. The motifs were considered over-represented if they had scores >1 and a p-value <1, where the p-value was generated against the background sequence sets. The upstream 1,000 bp regions of 88 *DEFL*s not expressed in nodules and 3,000 non-*DEFL* (Mt2.0) genes were used as background sets.

Correlation among the motif matrices from MEME versus the matrices of over-represented elements from TRANSFAC was calculated using the Pearson Correlation Coefficient column comparison metric of STAMP [34] (http://www.benoslab.pitt.edu/stamp). Similar parameters were used for statistical correlation of consensus sequences of *cis*-elements from the PLACE 30.0 database [35] nodule-specific elements against the matrices of MEME motifs.

### Generation and Evaluation of Promoter Deletions

Upstream segments of MtTC103606_at, MtTC95126_at, and MtTC100321_s_at were PCR-amplified from their respective BAC clones (primers and templates listed in Table S9) and ligated into pENTR-D/TOPO (Invitrogen). LR recombination was performed with the Gateway-compatible vector pKGW-R:EGFP-GUS, which is a modified pKGW-R plasmid [65] that includes the EGFP-GUS fusion gene. All plant expression vectors were transformed into *Agrobacterium rhizogenes* Arqua1 [66]. Transgenic hairy roots were generated using Medicago A17 seedlings as described previously [67], using 20 µg/mL kanamycin for selection. After formation of hairy roots (∼2.5 cm in length), all of the roots except one transgenic root, were removed and the plants were transferred to 0.5X Gamborg’s B5 Basal Salt medium (Sigma) with 1% plant tissue culture grade agar (Sigma) to recover from antibiotic selection. After 1 week, the plants were transferred to 2.25-inch square pots filled with Turface and inoculated with 100 µL/root of a washed suspension of Sm1021 cells (OD_600_ = 0.05) in sterile water.

The plants were screened 14 dpi for expression of *DsRED1* in the T-DNA using a Nikon SMZ 1500 microscope with DsRed filter set (EX 545, DM 570, BA 620). Nodules were harvested from roots positive for the DsRED marker and assayed for ß-glucuronidase (GUS) activity by infiltrating with 2 mM 5-bromo-4-chloro-3-indoxyl-ß-D-glucuronide cyclohexylammonium salt (X-Gluc), 0.1%Triton X-100, 50 mM NaPO_4_, pH 7.2, 2 mM potassium ferrocyanide, 2 mM potassium ferricyanide under vacuum for 30 min and incubating at 37°C overnight.

For confirmation of the GUS expression pattern, *in situ* hybridization of RNA corresponding to MtTC100321_s_at with nodule sections was performed. The coding region of MtTC100321_s_at was PCR-amplified (primers and templates listed in Table S9) and ligated into pGEMTeasy (Promega, Madison, WI) then cloned into the pBlueScriptKS+ vector (Stratagene, Santa Clara, CA) for digoxigenin (DIG) labeling. Linearized plasmid was used for *in vitro* transcription using DIG-11-UTP (Roche, Indianapolis, IN) and T7 and T3 polymerases. Nodules were harvested from roots 14 dpi that had been cultured on BNM as described above. Nodule fixation, sectioning, hybridization, and signal detection were carried out as described by Sbabou et al. [68].

### RNAi and Over-Expression Vector Construct Design and Plant Analyses

For RNAi constructs, 150 to 200 bp from the coding regions of five *NCR*s (MtTC100321_s_at, MtTC100264_at, MtTC94214_x_at, MtTC108430_at, and MtAW775198_at) was amplified from their corresponding EST cDNA clone (primer and template details in Table S9). A fragment from a human myosin gene (NT010393.16) was used as a control sequence. The PCR products were cloned into pENTR-D/TOPO (Invitrogen). LR recombination was performed with a modified pHellsgate8 [69].

For over-expression, the entire coding regions of the same five *NCR*s were amplified from their cDNAs with *Xba*I and *Bam*HI recognition sequences at 5′ and 3′ ends, respectively (primers and templates listed in Table S9). The PCR products were cloned into pENTR-D/TOPO (Invitrogen). The cDNA was excised using *Xba*I and *Bam*HI and ligated into the binary vector pILTAB381 [70] with the *NCR* coding sequence controlled by the *cassava vein mosaic virus* (CsVMV) promoter. The pILTAB381 vector with a CsVMV::GUS gene was used as the control.

The constructs were transformed into *A. rhizogenes* Arqua1 and used to generate composite transgenic plants as described above. Plants were inoculated with Sm1021 as described above and cultured on BNM. Plants to be assayed at 40 dpi were transferred to 2.25-inch square pots filled with Turface and fertilized with 0.5X BNM once per week. Plant height, leaf color, length, shape and color of the root and root hair, and nodule shape, number, size, distribution, and color were assessed at 0, 14, and 40 dpi. Significant (P<0.05) differences between plants with RNAi constructs or over-expression constructs and the control vector were determined by the Mann-Whitney *U* test. Transcript abundance in transgenic roots was measured by quantitative RT-PCR (qRT-PCR) assays (primers and templates listed in Table S9). Total RNA extraction procedures were as described above and first-strand cDNA was prepared from 2 µg of total RNA with the Superscript RT II kit (Invitrogen) and oligo dT primers (Sigma-Aldrich) at 200 ng/reaction, according to the manufacturer’s instructions. RT-PCR conditions were as described previously [71].

For evaluation of susceptibility to *Phytophthora medicaginis*, Sm1021-inoculated and mock-inoculated RNAi, over-expression, and vector control lines were transferred at 14 dpi to 2.25-inch square pots filled with Turface and inoculated with 1 mL *P. medicaginis* M2019 inoculum prepared as described previously [72]. Plants were flooded with sterile water and covered with a clear plastic dome for 2 days. The excess water and the covers were then removed and plants were fertilized with Peters Professional 10∶10:10 fertilizer (0.5X, Scotts) every 3 days. Disease symptoms were rated at 7 and 12 dpi [73].

## Supporting Information

Figure S1 **Acetylene reduction assay for determining the onset of nitrogen fixation of nodules at 6, 7, and 8 d post-inoculation (dpi).** Error bars indicate standard error.
(TIF)

Figure S2 **The five conserved motifs found in the upstream regions of** ***NCR*** **s.** The motifs were identified using MEME. These five motifs have the highest E-values of all motifs identified and are represented in more than half of the input *NCR* sequences.
(TIF)

Figure S3 **Summary of promoter deletion assays.** A, B and C are transgenic nodules with constructs for GUS expression of genes corresponding to MtTC103606_at, MtTC95126_at, and MtTC100321_s_at containing the (1) 400 bp, (2) 1,000 bp and (3) 2,000 bp to 400 bp upstream regions from the translation start site, respectively. Nodules were stained at 14 dpi for GUS activity.
(TIF)

Figure S4 **Comparison of the shape and number of bacteroids in nodules of transgenic and control lines.** A, Confocal image of a nodule section from an MtTC100321_s_at RNAi plant. B, Confocal image of a 14 dpi nodule section from a myosin RNAi (control). C, Rhizobia from a nodule of an MtTC100321_s_at RNAi plant. D, Rhizobia from a nodule of a myosin RNAi plant (control). E, Density plot showing the ratio of bacteria to bacteroids from an MtTC100321_s_at RNAi plant. F, Density plot showing the ratio of bacteria to bacteroids from a myosin RNAi plant (control).
(TIF)

Figure S5 **Real-time PCR verification of target gene expression in transgenic RNAi and over-expression lines.** Six transgenic plants from (A) RNAi and (B) over-expression lines corresponding to MtTC100321_s_at, MtTC100264_at, MtTC94214_x_at, MtTC108430_at, and MtAW775198_at were assayed for target gene expression using quantitative RT-PCR. Fold-change values are the ratio of the transgenic roots vs. the transgenic control roots at 14 dpi. Error bars indicate standard error of the three technical replicates.
(TIF)

Figure S6 **Real-time PCR verification of microarray data across different treatments.** The blue bars represent the fold-change values of MtTC100321_s_at in different treatments obtained from quantitative RT-PCR and the maroon bars represent corresponding fold-change values from microarray analysis. Values were calculated as treatment vs. mock-inoculated roots at 14 dpi except for mock-inoculated 14 dpi roots, where the relative expression was against mock-inoculated roots at 0 dpi. Error bars indicate standard error of the three biological replicates.
(TIF)

Table S1 **Differentially expressed** ***NCR*** **s in nodules at different developmental stages.**
(XLSX)

Table S2 **Differentially expressed** ***NCR*** **s in mock-inoculated roots.**
(XLSX)

Table S3 **Mean expression values of** ***NCR*** **s across treatments.**
(XLSX)

Table S4 **Differentially expressed** ***NCR*** **s in nodules inoculated with** ***S. meliloti*** **mutants.**
(XLSX)

Table S5 **Differentially expressed** ***NCR*** **s in nodules from inoculation with** ***bacA*** **at 14 dpi compared to nodules from inoculation with Sm1021 at 14 dpi.**
(XLSX)

Table S6 **Differentially expressed** ***NCR*** **s in nodules from inoculation with** ***dnf1*** **at 7 dpi and nodules from inoculation with** ***bacA*** **at 14 dpi compared to nodules from inoculation with Sm1021.**
(XLSX)

Table S7 **Upstream 1,000 bp sequence of 209 NCRs used to identify** ***cis*** **-element motifs.**
(XLSX)

Table S8 **Patterns of unique motifs in** ***NCR*** **promoters.**
(XLSX)

Table S9 **Primers and templates used in this research.**
(XLSX)
