# Supplementary figures and images for: Regulatory Patterns of a Large Family of Defensin-Like Genes Expressed in Nodules of Medicago truncatula
Source: PLoS One. 2013 Apr 1;8(4):e60355. doi: 10.1371/journal.pone.0060355 (PMC3613412; doi:10.1371/journal.pone.0060355)

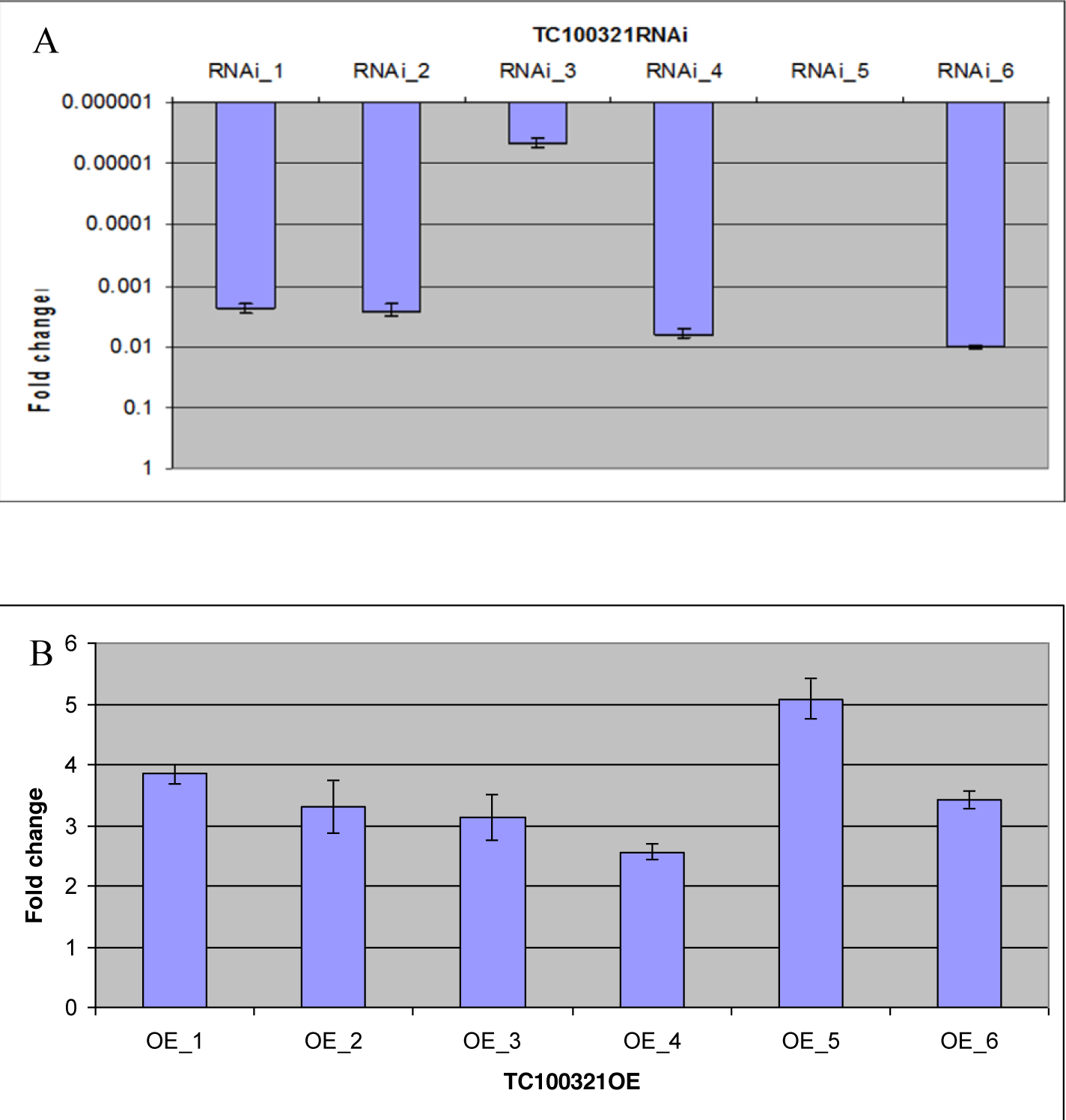

Supplement: Figure S1 — Acetylene reduction assay for determining the onset of nitrogen fixation of nodules at 6, 7, and 8 d post-inoculation (dpi). Error bars indicate standard error. (TIF) [file pone.0060355.s001.tif]

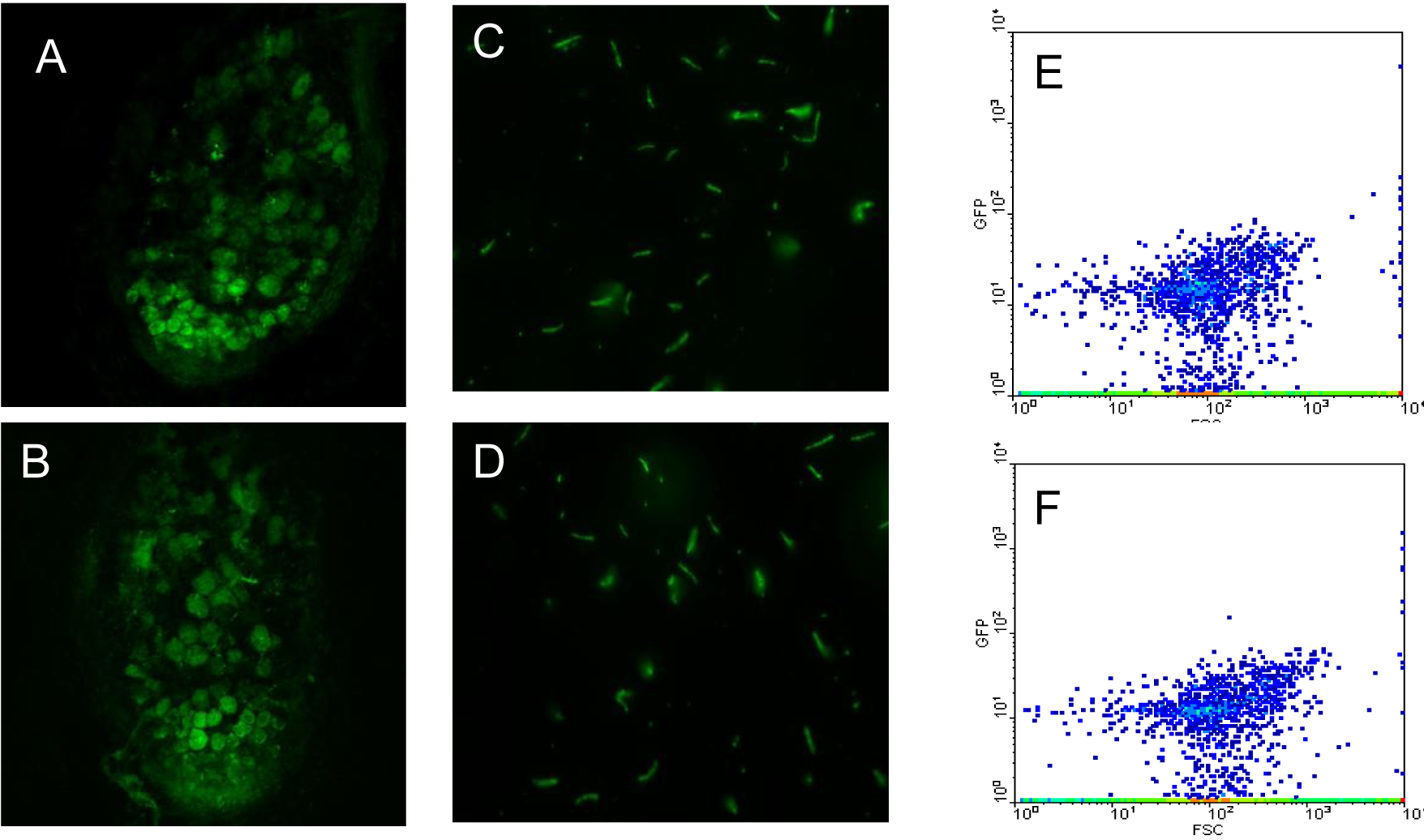

Supplement: Figure S2 — The five conserved motifs found in the upstream regions of NCR s. The motifs were identified using MEME. These five motifs have the highest E-values of all motifs identified and are represented in more than half of the input NCR sequences. (TIF) [file pone.0060355.s002.tif]

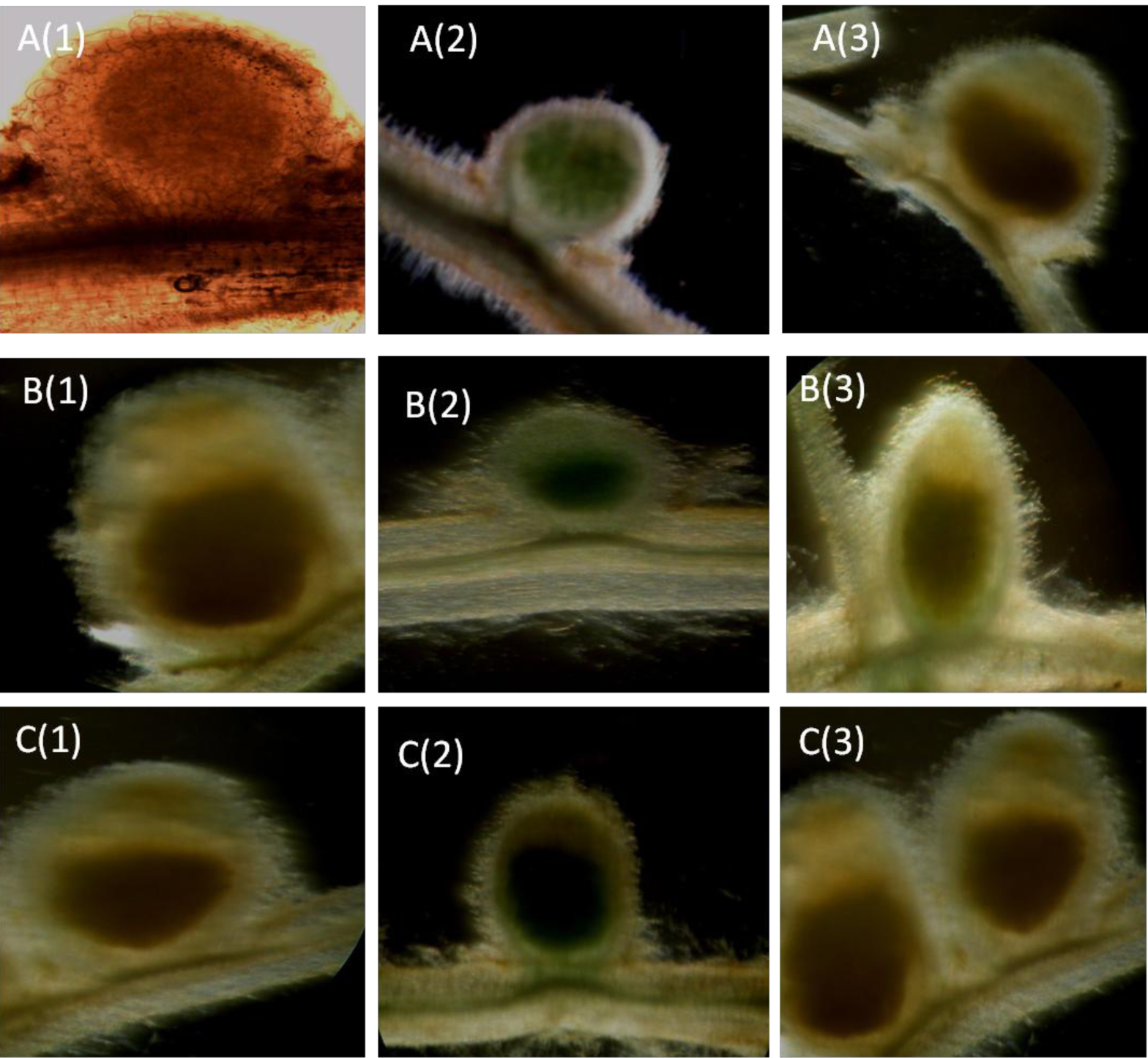

Supplement: Figure S3 — Summary of promoter deletion assays. A, B and C are transgenic nodules with constructs for GUS expression of genes corresponding to MtTC103606_at, MtTC95126_at, and MtTC100321_s_at containing the (1) 400 bp, (2) 1,000 bp and (3) 2,000 bp to 400 bp upstream regions from the translation start site, respectively. Nodules were stained at 14 dpi for GUS activity. (TIF) [file pone.0060355.s003.tif]

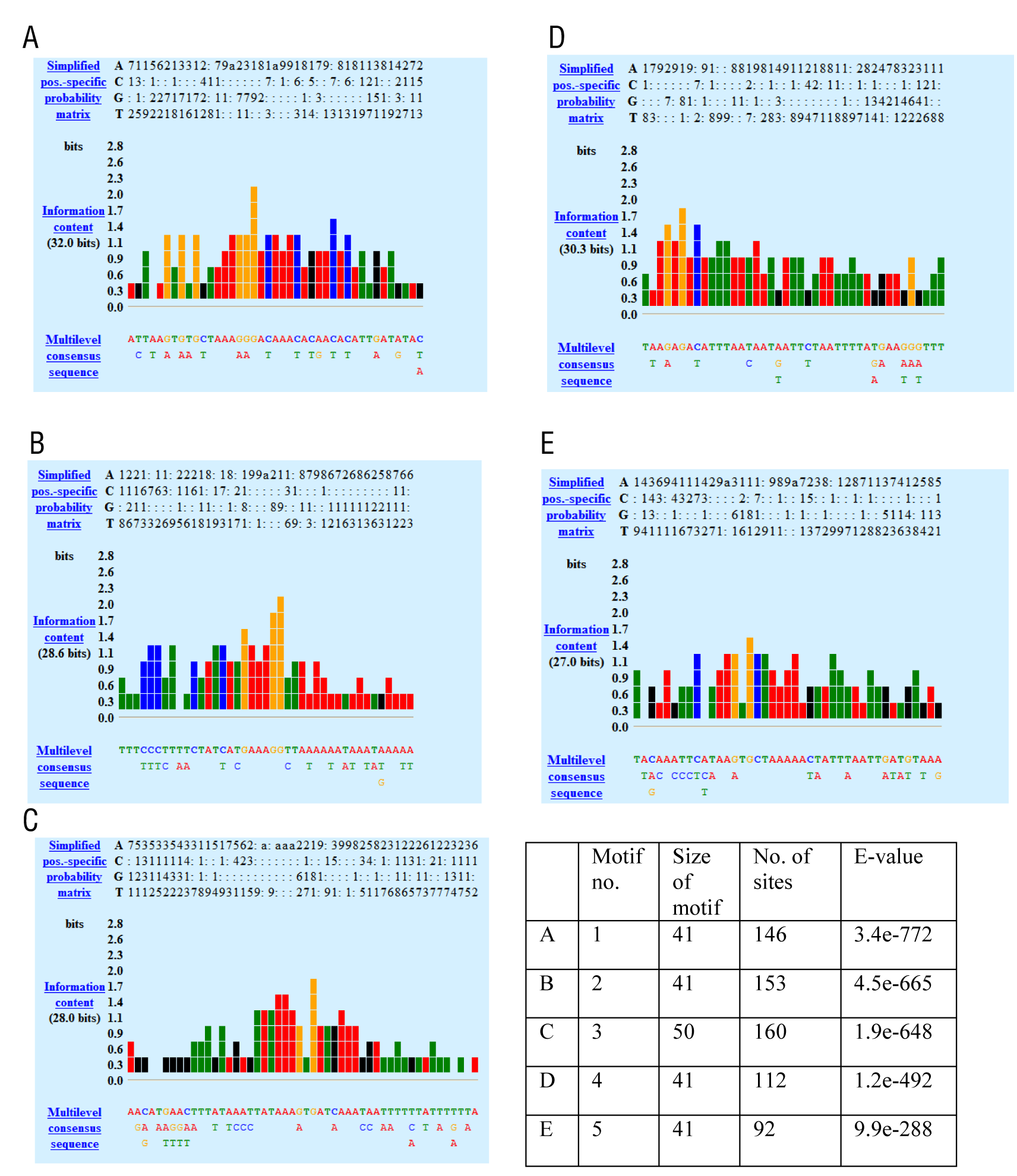

Supplement: Figure S4 — Comparison of the shape and number of bacteroids in nodules of transgenic and control lines. A, Confocal image of a nodule section from an MtTC100321_s_at RNAi plant. B, Confocal image of a 14 dpi nodule section from a myosin RNAi (control). C, Rhizobia from a nodule of an MtTC100321_s_at RNAi plant. D, Rhizobia from a nodule of a myosin RNAi plant (control). E, Density plot showing the ratio of bacteria to bacteroids from an MtTC100321_s_at RNAi plant. F, Density plot showing the ratio of bacteria to bacteroids from a myosin RNAi plant (control). (TIF) [file pone.0060355.s004.tif]

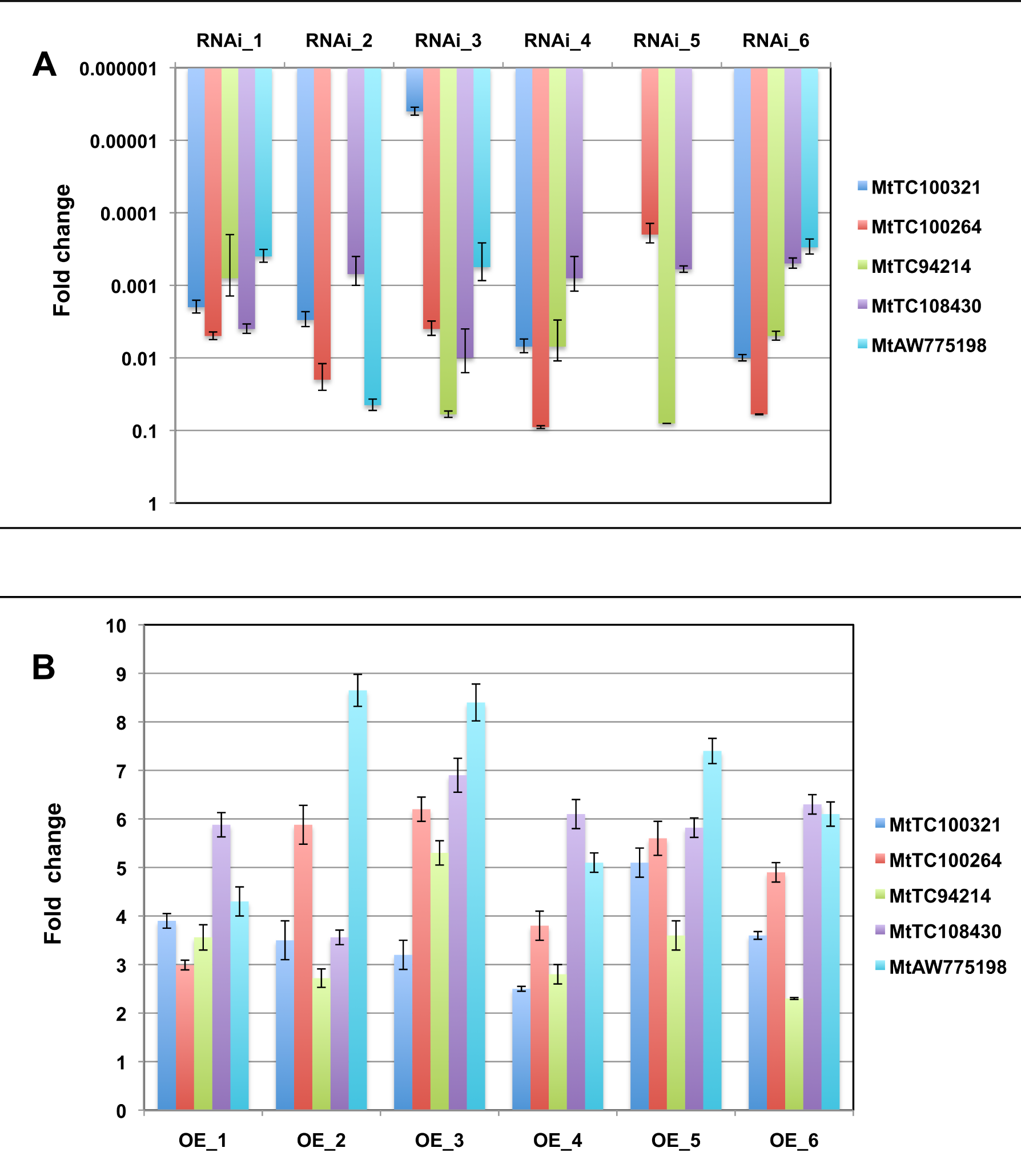

Supplement: Figure S5 — Real-time PCR verification of target gene expression in transgenic RNAi and over-expression lines. Six transgenic plants from (A) RNAi and (B) over-expression lines corresponding to MtTC100321_s_at, MtTC100264_at, MtTC94214_x_at, MtTC108430_at, and MtAW775198_at were assayed for target gene expression using quantitative RT-PCR. Fold-change values are the ratio of the transgenic roots vs. the transgenic control roots at 14 dpi. Error bars indicate standard error of the three technical replicates. (TIF) [file pone.0060355.s005.tif]

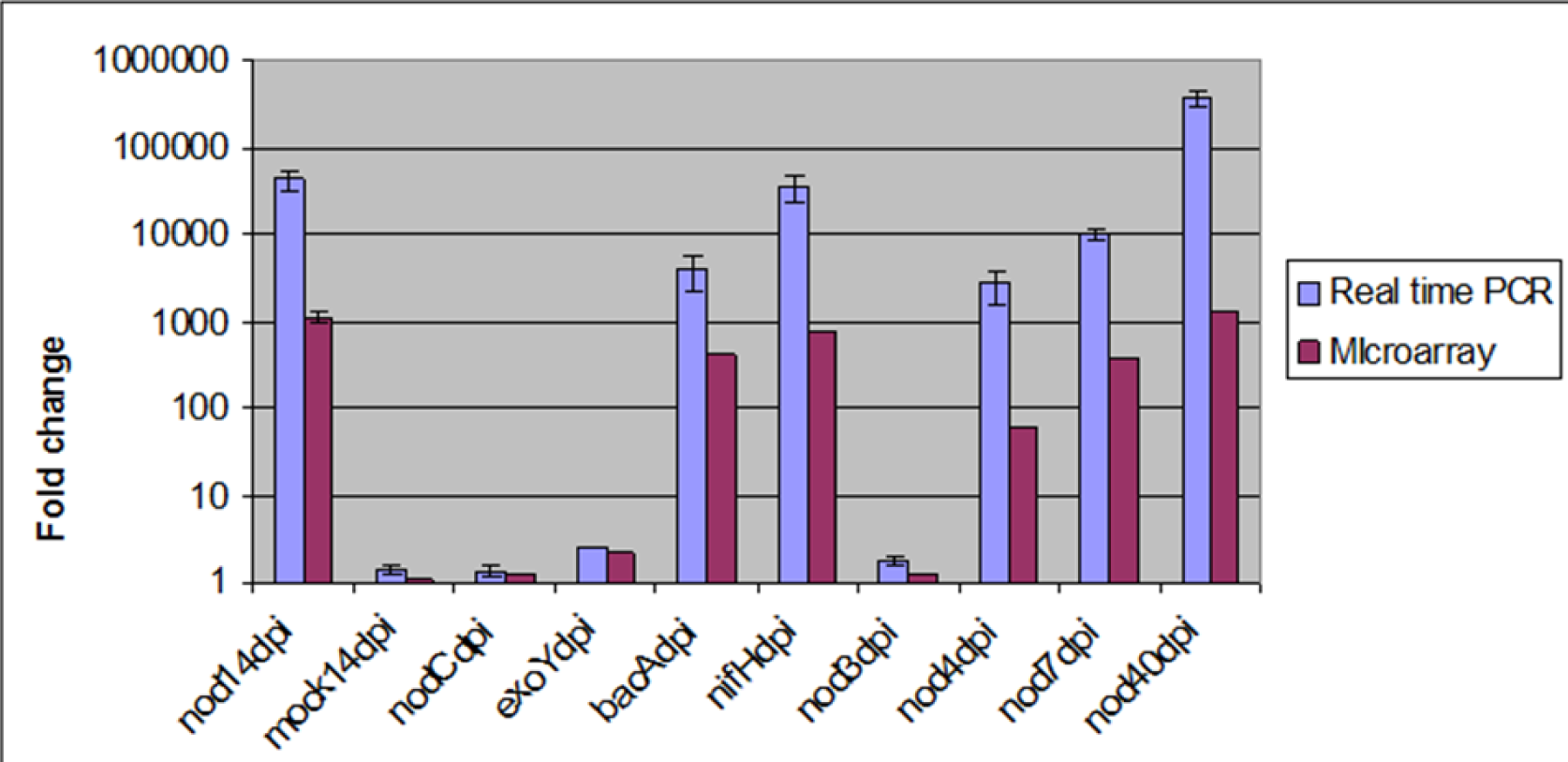

Supplement: Figure S6 — Real-time PCR verification of microarray data across different treatments. The blue bars represent the fold-change values of MtTC100321_s_at in different treatments obtained from quantitative RT-PCR and the maroon bars represent corresponding fold-change values from microarray analysis. Values were calculated as treatment vs. mock-inoculated roots at 14 dpi except for mock-inoculated 14 dpi roots, where the relative expression was against mock-inoculated roots at 0 dpi. Error bars indicate standard error of the three biological replicates. (TIF) [file pone.0060355.s006.tif]
